# Case Report: Treatment of Oral Lichen Planus With a Focus on Psychological Methods

**DOI:** 10.3389/fpsyt.2021.731093

**Published:** 2021-09-01

**Authors:** Xiuli Song, Xueqi Wu, Chunye Wang, Shuguang Sun, Xiangyang Zhang

**Affiliations:** ^1^Clinical Psychology, Yantai Affiliated Hospital of Binzhou Medical University, Yantai, China; ^2^Department of Psychology, University of Washington, Seattle, WA, United States; ^3^Department of Imaging, Yantai Mountain Hospital of Yantai City, Yantai, China; ^4^CAS Key Laboratory of Mental Health, Institute of Psychology, Chinese Academy of Sciences, Beijing, China

**Keywords:** oral lichen planus, major depressive disorder, psychogenic disease, psychological counseling, medication

## Abstract

Oral lichen planus (OLP) is one of the most common chronic diseases; however, its etiology remains unknown. More and more studies have revealed that emotional instability is one of the risk factors for the onset and expansion of OLP, especially in patients suffering from depression, anxiety disorder, and acute stress. In this case report, we had a 32-year-old female OLP patient who had no obvious response to conventional OLP drugs. Then we switched to a combination of psychotropic drugs and psychotherapies. By regulating mood through drugs and psychological counseling, the patient's oral disease was alleviated. Our case shows that clinicians should consider the mental problems of OLP patients. It also emphasizes the importance of medications and psychological counseling in the treatment of somatic diseases.

## Introduction

Oral Lichen Planus (OLP) was first recorded by Anderasen and Pindborg (1). OLP is a chronic inflammatory disease that affects the mucosa in the oral cavity. Currently, there are 6 types of oral lichen planus clinically: reticular (the most common type, showing fine white line at the lesion), atrophic, papular, bullous, plaque, and erosive types (2). OLP affects women by a 1.4:1 ratio over men (2). The origin and pathogenesis of OLP have been a hot topic for decades; however, it remains mysteriously unexplained. Therefore, the treatment methods vary. Many authors point out that there is a strong correlation between psychological factors (stress, anxiety, and depression) and the development of OLP (3, 4). Furthermore, scholars have argued that counseling sessions and stress management should be part of the OLP management protocol (2).

## Case Presentation

A 32-year-old female patient was referred to the Department of Clinical Psychology due to severe affective symptoms in the past 4 months. Eight years ago, the onset of her symptoms was triggered by the extreme unpleasantness between the patient and her mother. Heteroanamnestic revealed that the patient grew up in a divorced family, and then lived with her mother afterwards. Her mother often abused her verbally. The fierce quarrels reached peak 4 months ago. After that, the patient began to develop affective and psychosomatic symptoms, including poor sleep quality, anhedonia, loss of appetite, stomach pain, and fatigue. Therefore, she was hospitalized for further observation due to depression. The medical history of the patient revealed that she was diagnosed with OLP 5 months before this referral. As stated by the patient, 9 months ago, she suffered from discomfort when chewing and speaking. Although the patient thought that it was an aphthous ulcer, she did not receive any treatment. However, after a few months, as the tension between the patient and her mother deteriorated, the patient began to develop more severe symptoms, including a burning sensation in the oral cavity and increased sensitivity to spicy and hot foods. She then reported that she went to Beijing Stomatological Hospital and was diagnosed with OLP (5). The stomatologist prescribed budesonide, vitamin B2, cetylpyridnium chloride, and advised her to visit the Department of Clinical Psychology. The patient reported that she did not feel better after taking traditional OLP drugs, and later forget the detailed information about OLP drugs, such as what the type and dose of Budesonide was, and whether other corticosteroid drugs such as triamcinolone was used. In addition, the patient denied having parafunctional habits such as bruxism or clenching.

The patient neither smoked nor drank. She was single and had no history of pregnancy. Her family members disclosed no mental illness. During the mental status examination, the patient looked tired and pale, and she could not help crying. The patient had a flat affect when answering questions. She spoke slowly. Cognitive function was very good. The patient's behaviors and facial expressions showed low mood and moderate anxiety. In the subsequent psychological assessments, including the Minnesota Multiphasic Personality Inventory-2 (MMPI-2), Self-Rating Depression Scale (SDS) and Self-Rating Anxiety Scale (SAS), the results revealed that she had relatively high levels of depression and anxiety (scores: SDS = 65, SAS = 70).

Her vital signs were stable, and no abnormal physical or neurological signs were detected at admission. The patient's blood biochemical results were all within the normal range, including blood cell analysis, C-reactive protein (CRP), hepatic function, renal function, infection markers, the quantitation of HBV markers, thyroid function, prolactin, sex hormone, alexin (C4), antinuclear antibodies, antinuclear extract antibody spectrum determination, folic acid, vitamin B12, trace elements, procalcitonin (PCT), and cortisol. However, alexin (C3: 0.8 g/L, normal: 0.9–1.8 g/L) was decreased. Also, the test results of urine sediment quantification, urine analysis, fecal analysis, and occult blood analysis were all within the normal range. Electroencephalogram (EEG) and electrocardiograph (ECG) showed no abnormalities; however, during the first brain MRI examination, the pituitary signal was found to be uneven. Seven days later, the second examination of pituitary dynamic enhanced MRI scan showed no abnormality. During the hospitalization, the patient went to consult the stomatology doctor of our hospital to reconfirm diagnose of OLP. The result of consultation was that there were white plaques on bilateral buccal mucosa, upper and lower mandibular vestibular sulcus mucosa, and a small amount of reticulation in the buccal sulcus, which could not be removed. There were ulcers and erosions in the white metamorphic areas of the vestibular sulcus, superficial, and tenderness. Taken together, the diagnosis was reconfirmed to be oral lichen planus. Because the patient's oral ulcers were severe, the stomatologist did not recommend pathological and immunological examinations, nor recommend changing medications. According to the International Classification of Diseases (ICD-10), she was diagnosed with a depressive episode when she came to our hospital. The initial treatment was Venlafaxine (75 mg, every morning), Lorazepam (0.5 mg, every morning and every night), Shugan jieyu capsules (0.72 g, every morning and every night), together with the drugs for the treatment of OLP. But the drugs for the treatment of OLP was discontinued a week after admission.

Consultation meetings were arranged once a week, mainly using psychoanalytic methods to resolve inner conflicts with her mother, and cognitive behavior therapy (CBT) to adjust her emotions toward certain people and events. The effect was remarkable. A week and a half later, the patient reported that her mood was elevated and she barely cried. Moreover, the abdominal pain disappeared, and the burning and tightening sensation in the oral cavity gradually subsided.

After 1 month of follow-up, the patient's symptoms were significantly reduced, indicating that the psychotherapy had a good effect, and she continued to adhere to psychological counseling twice a week. The drug dose was gradually reduced. Lorazepam and Shugan jieyu capsules were discontinued, and only venlafaxine (75 mg, every morning) was taken. No obvious drug and psychological side effects were observed. The assessment results revealed relatively low levels of depression and anxiety (scores: SDS = 50, SAS = 48) (Table 1).

**Table 1:** Patient's demographic and clinical characteristics.

**Characteristics**	**Description**
Gender	Female
Age	32
Marriage	Single
Occupation	Employed
Illness Duration	8 years
Hospitalization times	1
Physical illness	Oral lichen planus
Drug Usage	Venlafaxine (75 mg, every morning), Lorazepam (0.5 mg, every morning and every night), Shugan jieyu capsules (0.72 g, every morning and every night), budesonide, vitamin B2, cetylpyridnium chloride
Frequency of psychotherapy	Once a week or twice a week
**Psychological evaluation during hospitalization**	
SDS	65
SAS	70
**Psychological evaluation during follow-up**	
SDS	50
SAS	48

*SDS, Self-Rating Depression Scale; SAS, Self-Rating Anxiety Scale*.

## Discussion

Although the etiology of OLP is unknown, we witnessed a tremendous recovery in patient's oral and mental conditions treated with psychotropic medications and multiple psychotherapeutic methods. Based on previous clinical studies, it is generally believed that mental stress is one of the possible risk factors for the development of OLP (6). A previous study showed that in postmenopausal women, stress could lead to anxiety and depression, which are common reasons of lichen planus (7). A study showed that 48% of the 274 OLP patients had mental problems, and anxiety, depression and stress existed in 45% of these OLP patients with mental problems (3). In other studies, OLP group had much higher anxiety, depression, and stress (8, 9). Taken together, these studies indicate that stress, anxiety, and depression may be the cause of OLP, and also the cause of OLP relapses.

Many authors believe that among patients with mental disorders, such as depression or psychological stress, changes in the immune system may also lead to OLP (6). In this case, due to insignificant effect after taking conventional drugs for OLP, we suspected that intense emotions may play a key role in her prolonging symptoms. Therefore, we added psychotropic drugs to strengthen the treatment, together with psychotherapies. In the end, the patient's condition responded well. The oral, psychosomatic, and mental symptoms gradually alleviated. In short, although we cannot verify the causal relationship between psychological factors and OLP, we had successfully used psychiatric drugs and psychotherapies to control OLP, when traditional treatments first failed.

### Medications

Current OLP therapy aims to eliminate all mucosal-related lesions, reduce symptoms and decrease the risk of oral cancer, including corticosteroids, immunomodulatory agents, retinoids, ultraviolet irradiation, and/or laser therapy (10). Topical steroids are still the first-line treatment for symptomatic OLP (11). In our study, the above-mentioned drugs were also used in this case, but the effect was not satisfactory. We added antidepressants and anti-anxiety drugs. During the hospital stay, the patient mainly used lorazepam and venlafaxine to relieve her depressive symptoms. In one previous study, benzodiazepine and antidepressant drugs were recommended as the best options for patients with oral lichen planus (12). In another study, psychotropic drugs proved to be effective in treating burning mouth syndrome (BMS)-like oral symptoms in patients with reticular oral lichen planus (R-OLP), who were refractory to conventional immunosuppressive therapy (13). In the previous study, 28 symptomatic R-OLP patients were given benzodiazepine and antidepressant drugs for 6 months. The results showed that the Hamilton depressive scale (HAMD) and the Hamilton anxiety scale (HAMA) scores improved significantly (13). As a receptor agonist, benzodiazepine can enhance the expression of the neurotransmitter gamma-aminobutyric acid (GABA), thereby leading to sleep induction, relaxation, and anti-anxiety. There are two main types of antidepressants, selective serotonin reuptake inhibitors (SSRIs) and serotonin and norepinephrine reuptake inhibitors (SNRIs). Venlafaxine is one of the SNRIs that inhibits the reuptake of both serotonin (5-HT) and norepinephrine (NE), thereby increasing the levels of 5-HT and NE in the brain, and alleviating depression. Therefore, we prescribed the widely-used benzodiazepine-lorazepam and SRNIs- venlafaxine for this patient.

### Psychotherapy

Many authors recommend various psychotherapies to prevent oral lichen planus (14, 15). In this case, we used CBT and psychodynamic methods to help the patient. Depression is a state of decreased psychophysical activities, characterized by predominate sadness and a slowdown in pessimistic thinking (16). CBT has been found to be very effective in managing chronic pain and insomnia (17). Wilfred and his colleagues found that CBT intervention significantly improved people with depressive symptoms, fatigue, and lack of sleep (18). In this case, sleep conditions were improved, and fatigue gradually disappeared after the fifth session.

On the other hand, this case fully demonstrated Freud's theory, that is, unresolved conflicts between mother and child can have a great impact on the consequences of abnormal mental health in future (19). Psychodynamically speaking, depression can also be interpreted as an auto-aggression. When libido begins to retreat from the lost goal to one's ego, aggression has already conquered the ego; this is why a depressed individual strives for self-destruction (16). Using the methods of psychoanalysis, the psychotherapist resolved the unresolved conflicts, and the patient could live more fulfilling lives. In addition to the strong correlation, Manolache and his colleagues found that among the three types of stressful events, family affairs have a special role in the extension of lichen planus lesions (20). In this study, the patient's stressful situation was also consistent with the pattern of Manolache's finding. After a conflict with her mother, the patient's oral symptoms deteriorated. Therefore, the patient received psychoanalytic counseling sessions to resolve their internal conflicts with herself and her mother.

In this case, the patient had two problems. First, the original family of the patient had problems. For example, the patient's parents' marriage was not harmonious, which led to insufficient care of the patient. She lacked security and independence, and developed dependent personality characteristics. Second, because of the patient's dependent personality characteristics, she relied too much on her boyfriend spiritually, which led to the unequal psychological status of the two parties in the emotional life, and at the same time brought too much mental pressure to the other party, resulting in a bad relationship and a strong emotional reaction between the patient and her husband.

Psychological therapies included, but not limited to: (1) guiding the patient to speak out and vent her emotions; (2) changing the patient's cognition, such as accepting her native family first, and then encouraging her self-growth and cultivating her independent personality; (3) asking the patient to accept her love and establish a healthy relationship with her boyfriend. Before the consultation, the patient was emotional, cried, and had a naive personality. She attributed her personal problems to her parents, boyfriend, and others. After treatment, the patient's mood gradually stabilized, admitted her personality problems, and at the same time believed that she needed to grow up and maintain healthy lifestyle.

In this case, the effect of psychotherapy is confirmed on the patient. After psychotherapy, the patient understood the influence of her own experience on her, learned how to get along with her mother and others, and can better deal with problems in daily life or work. However, the patient dared not stop the drug, and worried about the recurrence of the disease after stopping the drug. In the process of psychotherapy, the problem of drug withdrawal was also discussed, and the patient was encouraged to try to stop the drug. The patient still wanted to continue taking the drug for a period of time. Here, we respected the patient's selection. Perhaps psychotherapy will also take some time, and the psychologist continues to accompany the patient.

## Limitation

There were two main limitations. First, the clinical description of the OLP lesions, including the type of lesions, location, and severity of the lesion according to an accepted criterion such as “Thongprasom,” and the severity of pain and burning symptoms according to the VAS should be thoroughly compared before, after treatment and after follow-up. It should be best to have images of the OLP lesions for each stage. However, unfortunately, we did not record the specific conditions of the OPL lesions in detail due to the limit of the subjective conditions in our clinical practice. Second, in this treatment, we did not evaluate the efficacy of drugs and psychological counseling separately, but only evaluated the overall efficacy of the treatment after a period of time. We will evaluate the effects of drugs and psychological therapy separately for other clinical patients.

## Conclusion

In this case report, we witnessed and emphasized the importance of using a combination of psychiatric drugs and psychotherapies to treat emotion-related OLP. We believe that psychotropic drugs can enhance the patient's brain function, and psychotherapies can relieve stress, enabling patients to have a better quality of life in the future. From the current clinical evidence, the correlation between psychological factors and OLP is strong; however, the causality has not been verified. In the future, we need to better understand the etiology of OLP, so more research needs to be done so that OLP patients can efficiently obtain the most direct guidance and effective treatment.

## Data Availability Statement

The original contributions presented in the study are included in the article/supplementary material, further inquiries can be directed to the corresponding author/s.

## Ethics Statement

Written informed consent was obtained from the individual(s) for the publication of any potentially identifiable images or data included in this article.

## Author Contributions

XS, XW, and SS treated this patient and wrote the paper. CW contributed to the manuscript preparation. XS and XZ critically reviewed the diagnostic results and proofreading the manuscript. All authors read and approved the final version of the manuscript.

## Conflict of Interest

The authors declare that the research was conducted in the absence of any commercial or financial relationships that could be construed as a potential conflict of interest.

## Publisher's Note

All claims expressed in this article are solely those of the authors and do not necessarily represent those of their affiliated organizations, or those of the publisher, the editors and the reviewers. Any product that may be evaluated in this article, or claim that may be made by its manufacturer, is not guaranteed or endorsed by the publisher.

## Abbreviations

OLP: Oral lichen planus
MMPI-2: the Minnesota Multiphasic Personality Inventory-2
SDS: Self-Rating Depression Scale
SAS: Self-Rating Anxiety Scale
CRP: C-reactive protein
PCT: procalcitonin
ECG: Electrocardiograph
EEG: Electroencephalogram
ICD-10: the International Classification of Diseases
BMS: burning mouth syndrome
R-OLP: reticular oral lichen planus
HAMD: the Hamilton depressive scale
HAMA: the Hamilton anxiety scale
GABA: the gamma-aminobutyric acid
SSRIs: selective serotonin reuptake inhibitors
SNRIs: serotonin and norepinephrine reuptake inhibitors
5-HT: serotonin
NE: norepinephrine
CBT: Cognitive Behavioral Therapy.

## References

[1] Andreasen JO. Oral lichen planus. 1. A clinical evaluation of 115 cases. Oral Surg Oral Med Oral Pathol. (1968) 25:31–42. 5235654 10.1016/0030-4220(68)90194-1

[2] Sandhu SV Sandhu JS Bansal H Dua V. Oral lichen planus and stress: an appraisal. Contemp Clin Dent. (2014) 5:352–6. 10.4103/0976-237X.137946 25191072 PMC4147812

[3] Cassol-Spanemberg J Blanco-Carrion A Rodriguez-de Rivera-Campillo ME Estrugo-Devesa A Jane-Salas E Lopez-Lopez J. Cutaneous, genital and oral lichen planus: a descriptive study of 274 patients. Med Oral Patol Oral Cir Bucal. (2019) 24:e1–7. 10.4317/medoral.22656 30573709 PMC6344000

[4] Cerqueira JDM Moura JR Arsati F Lima-Arsati YBO Bittencourt RA Freitas VS. Psychological disorders and oral lichen planus: a systematic review. J Investig Clin Dent. (2018) 9:e12363. 10.1111/jicd.12363 30270524

[5] van der Meij EH van der Waal I. Lack of clinicopathologic correlation in the diagnosis of oral lichen planus based on the presently available diagnostic criteria and suggestions for modifications. J Oral Pathol Med. (2003) 32:507–12. 10.1034/j.1600-0714.2003.00125.x 12969224

[6] Gavic L Cigic L Biocina Lukenda D Gruden V Gruden Pokupec JS. The role of anxiety, depression, and psychological stress on the clinical status of recurrent aphthous stomatitis and oral lichen planus. J Oral Pathol Med. (2014) 43:410–7. 10.1111/jop.12148 24450470

[7] Sen S Sen S Dutta A Abhinandan Kumar V Singh AK. Oral manifestation and its management in postmenopausal women: an integrated review. Prz Menopauzalny. (2020) 19:101–3. 10.5114/pm.2020.97867 32802020 PMC7422290

[8] Shaw H Konidena A Malhotra A Yumnam N Farooq F Bansal V. Psychological status and uric acid levels in oral lichen planus patients - A case- control study. Indian J Dent Res. (2020) 31:368–75. 10.4103/ijdr.IJDR_289_19 32769269

[9] Zucoloto ML Shibakura MEW Pavanin JV Garcia FT da Silva Santos PS Maciel AP. Severity of oral lichen planus and oral lichenoid lesions is associated with anxiety. Clin Oral Investig. (2019) 23:4441–8. 10.1007/s00784-019-02892-2 30989337

[10] Rotaru D Chisnoiu R Picos AM Picos A Chisnoiu A. Treatment trends in oral lichen planus and oral lichenoid lesions (Review). Exp Ther Med. (2020) 20:198. 10.3892/etm.2020.9328 33123228 PMC7588785

[11] Oberti L Alberta L Massimo P Francesco C Dorina L. Clinical management of oral lichen planus: a systematic review. Mini Rev Med Chem. (2019) 19:1049–59. 10.2174/1389557519666190301144157 30836913

[12] Guarneri F Guarneri C Marini H. Oral lichen planus and neurogenic inflammation: new observations and therapeutic implications from four clinical cases. Dermatol Ther. (2014) 27:206–10. 10.1111/dth.12118 24548522

[13] Adamo D Mignogna MD Pecoraro G Aria M Fortuna G. Management of reticular oral lichen planus patients with burning mouth syndrome-like oral symptoms: a pilot study. J Dermatol Treat. (2018) 29:623–9. 10.1080/09546634.2018.1425359 29308937

[14] Vilar-Villanueva M Gándara-Vila P Blanco-Aguilera E Otero-Rey EM Rodríguez-Lado L García-García A. Psychological disorders and quality of life in oral lichen planus patients and a control group. Oral Dis. (2019) 25:1645–51. 10.1111/odi.13106 30993798

[15] Parlatescu I Tovaru M Nicolae CL Sfeatcu R Didilescu AC. Oral health-related quality of life in different clinical forms of oral lichen planus. Clin Oral Investig. (2020) 24:301–8. 10.1007/s00784-019-02951-8 31098713

[16] Pokupec JS Gruden V Gruden V Jr. Lichen ruber planus as a psychiatric problem. Psychiatr Danubina. (2009) 21:514–6. 19935485

[17] Alrashdan MS Alkhader M. Psychological factors in oral mucosal and orofacial pain conditions. Eur J Dent. (2017) 11:548–52. 10.4103/ejd.ejd_11_17 29279685 PMC5727744

[18] Pigeon WR Funderburk J Bishop TM Crean HF. Brief cognitive behavioral therapy for insomnia delivered to depressed veterans receiving primary care services: a pilot study. J Affect Disord. (2017) 217:105–11. 10.1016/j.jad.2017.04.003 28395207

[19] Yaacob MJ. Parent-adolescent relationships and its association to adolescents' self-esteem. The Malays J Med Sci. (2006) 13:21–4. 22589586 PMC3347898

[20] Manolache L Seceleanu-Petrescu D Benea V. Lichen planus patients and stressful events. J Eur Acad Dermatol Venereol. (2008) 22:437–41. 10.1111/j.1468-3083.2007.02458.x 18363912

